# Plant-Based Bioinsecticides for Mosquito Control: Impact on Insecticide Resistance and Disease Transmission

**DOI:** 10.3390/insects13020162

**Published:** 2022-02-03

**Authors:** Meryem Ş. Şengül Demirak, Emel Canpolat

**Affiliations:** Department of Molecular Biology and Genetics, Tokat Gaziosmanpaşa University, Tokat 60150, Turkey; emel.canpolat@gop.edu.tr

**Keywords:** bioinsecticide, disease transmission, insecticide-resistance, mosquito-borne disease, mosquito control, natural compounds, phytochemical

## Abstract

**Simple Summary:**

Mosquito-borne diseases cause millions of deaths each year. There has been an increase in the use of insecticides to combat disease transmission caused by mosquitoes. Synthetic insecticides have been effectively used to protect humans from mosquito bites through insecticide-treated mosquito nets, fabrics, and indoor sprays. Despite the considerable progress made in reducing mosquito borne diseases, extensive usage of insecticides has caused serious health problems to humans and animals, insecticide resistance or insensitivity in mosquitoes, and environmental damage. A success in the fight with mosquito disease transmission can only be accomplished by adequate and effective implementation of insecticide resistance monitoring and management programs globally. For this purpose, extensive research focuses on exploring insecticide resistance mechanisms in mosquitoes and how they get resistant to chemical applications over time. The search also focuses on novel compounds that are more effective, safer, and eco-friendly for improved management of mosquito vectors. In this review, we provide the current literature on the synthetic insecticides and how mosquitoes develop resistance to them, with further emphasis on bioinsecticides that could replace conventional synthetic insecticides. In this context, plant-based compounds are explained in detail with their potential applications to control mosquitoes.

**Abstract:**

The use of synthetic insecticides has been a solution to reduce mosquito-borne disease transmission for decades. Currently, no single intervention is sufficient to reduce the global disease burden caused by mosquitoes. Problems associated with extensive usage of synthetic compounds have increased substantially which makes mosquito-borne disease elimination and prevention more difficult over the years. Thus, it is crucial that much safer and effective mosquito control strategies are developed. Natural compounds from plants have been efficiently used to fight insect pests for a long time. Plant-based bioinsecticides are now considered a much safer and less toxic alternative to synthetic compounds. Here, we discuss candidate plant-based compounds that show larvicidal, adulticidal, and repellent properties. Our discussion also includes their mode of action and potential impact in mosquito disease transmission and circumvention of resistance. This review improves our knowledge on plant-based bioinsecticides and the potential for the development of state-of-the-art mosquito control strategies.

## 1. Introduction

Mosquitoes have been a big burden to human health for a long time. These insects can invade in different geographic locations and new habitats through global trade and travel [1] which causes millions of people be at risk of the diseases they transmit. In 2019, an estimated 229 million cases and 409 thousand deaths for malaria and 56 million cases for dengue have been reported worldwide [2,3]. While malaria case incidences were reported to decline, the number of malaria endemic countries has increased in the period 2000–2019 [2]. The global incidence of dengue is thought to be increased about thirty times over the last fifty years with emergencies in new countries [4,5,6]. A recent study also indicates that mosquito species will continue to spread globally over the coming decades, which may cause about 50% of the world’s population at the risk of mosquito-borne viral disease transmission by 2050 [7]. Even a more serious problem is at our doorstep as the climate change is expected to increase the burden of mosquito-borne diseases despite the ongoing disease control interventions [8,9].

The most common way of keeping mosquitoes away from their human hosts is to use synthetic insecticides in mosquito nets, fabrics, and indoor sprays. The usage of chemical strategies has brought hope in controlling disease transmission in endemic regions, but emergence of insecticide resistance has been a major problem in reducing the disease burden. The uncontrolled usage of insecticides has led to reemergence and increase in mosquito populations over the years. Between the years 2010–2019, about 28 malaria endemic countries (out of 82) have detected resistance to all four classes of the most commonly used insecticides, and 73 have detected resistance to at least one insecticide class, an issue that continues to increase globally [2]. Thus, insecticide resistance is now considered a serious threat to control mosquito invasion and disease transmission. It is essential that the methods for insecticide monitoring in mosquito populations and interpretation of results are performed adequately, effectively and in a timely manner for improving mosquito control [10,11]. 

Current research on mosquito control is now focused on understanding the mosquito resistance to synthetic insecticides and developing novel strategies to overcome the resistance issues. Natural compounds that are more effective and less toxic than the synthetic ones continue to get more attention in the research community. The use of bioinsecticides, composed of botanical or plant-based compounds, has been a perfect alternative due to their minimal hazardous effects on human health and environment. In this review, we provide current knowledge on synthetic insecticides that are actively used in mosquito control and how they impact prevalence of insecticide resistance in mosquitoes. Major plant-based insecticides, their mode of action and the research about their potential mosquitocidal activity are discussed. A comprehensive understanding of how biochemical compounds can be advantageous to synthetic ones and how we can circumvent insecticide resistance issues in the fight with mosquito-borne disease transmission is provided. 

## 2. Insecticide-Based Mosquito Control Strategies

Insecticide-based mosquito control plays an important role in efforts to reduce the transmission of mosquito-borne diseases worldwide. Two core insecticidal interventions are in use to control mosquitoes: deployment of insecticide-treated mosquito nets (ITNs) and indoor residual spraying (IRS) of insecticides [10]. These interventions have been effectively used to kill mosquitoes or interfere with their host-seeking behavior to prevent disease transmission worldwide [12,13,14,15,16,17,18,19,20]. The global malaria cases and malaria death rates have declined about 18% and 48%, respectively, between the years 2000 and 2015, and 70% reduction in malaria cases in sub-Saharan Africa was attributed to ITNs, and 10% reduction was due to IRS [21].

**Figure 1:**
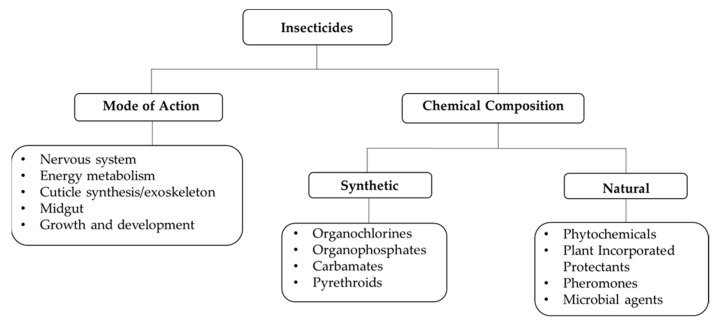
Classification of insecticides based on mode of action and chemical composition.

Four classes of insecticides are mostly used in mosquito control programs which include pyrethroids (e.g., deltamethrin, permethrin, cypermethrin, lambda-cyhalothrin), organochlorines (e.g., DTT), organophosphates (e.g., malathion, fenitrothion), and carbamates (e.g., propoxur, bendiocarb) [10] (Figure 1). Most synthetic insecticides have physiological or behavioral impact on mosquitoes (Figure 1), and predominantly target the central nervous system of insects. Among them, pyrethroids are the most widely used insecticides for IRS and the only synthetic insecticide currently used in ITNs and fabrics, with irritant or repellent activity on mosquitoes and less mammalian toxicity [2]. They disrupt the voltage-gated sodium channels in neuronal membranes [22]. When pyrethroids bind an open channel, they prevent its closure, thus leading to a prolonged action potential or disruption of electrical signaling in the nervous system [23,24,25]. This causes continuous nerve excitation and paralysis (or knockdown) of the insect and eventually its death [26]. 

While pyrethroids have been effectively used in ITNs to control mosquitoes for a long time, prevalence of pyrethroid resistance in mosquito species causes a major problem to combat disease transmission worldwide [27,28,29]. Like pyrethroids, some organochlorines are also inhibitors of the insect’s voltage-gated sodium channels. Dichlorodiphenyltrichloroethane (DDT) is an example that targets sodium channels, and it is the first and the most commonly used synthetic insecticide of organochlorine in residual spraying. Its low cost and high effectiveness have made it a favorable chemical for indoor wall spraying. However, resistance developed to DDT in various mosquito species and its toxic effects on humans and non-target organisms have imposed limitations or restrictions in its usage [30,31]. Other organochlorines (such as cyclodienes, dieldrin and fipronil) target γ-amino butyric acid (GABA) receptors, which are hetero-multimeric gated chloride channels in the insect’s central nervous system [32]. Cyclodiene insecticides act as neurotoxicants and block the GABA receptors causing hyper-excitation of the central nervous system, convulsions, and eventually death of insects [33,34,35]. Organophosphates (OP) and carbamates are two other insecticides sharing similar mode of action. They inhibit acetylcholinesterase (AChE) enzyme, preventing breakdown of the neurotransmitter acetylcholine, resulting in neuromuscular overstimulation and death of insects [36,37,38]. Due to pyrethroid and DDT resistance issues worldwide, they have been used as alternative insecticides in IRS, but they have a shorter residual effectiveness, high toxicity to mammals, and are more costly compared to the others that limit their persistent long-term usage. 

## 3. Insecticide Resistance in Mosquitoes

Short after its first usage in California in 1945, the resistance of mosquitoes to DDT was reported [39,40]. Since then, insecticidal resistance in mosquitoes has been reported, with a substantial increase between 2010 and 2016 [10]. In these years, insecticide resistance was found to be widespread in *Anopheles* vectors in malaria endemic African regions and insecticide resistance frequency has changed over time [10]. Understanding pyrethroid resistance development in *Anopheles* mosquitoes is particularly important because its prevalence can disable pyrethroid-treated ITN-based interventions, which are used successfully for malaria control [41,42]. Pyrethroid resistance was determined to be very high in the WHO African Region (78%), Eastern Mediterranean Region (70%), and in the South-East Asia Region (38%), Western Pacific Region (51%), but was lower in the Region of the Americas (20%). The incidence of organochlorine resistance was also similar in all WHO regions (60–70%). Carbamate resistance prevalence was between 22% and 54%, and organophosphate resistance prevalence varied widely across regions, 14% in the WHO African Region and 65% in the WHO Western Pacific Region [10]. While resistance frequencies are generally high in most of the endemic regions, those with lower resistance frequencies could be an indication of recent gain of resistance or selection for resistant populations to insecticides [43].

Despite effective use of insecticide-based mosquito control strategies for decades, their prolonged usage is challenged by high cost, toxicity and, more importantly, the development of resistance to the synthetic insecticides. Insecticide resistance is mostly inferred to the ability of insects to survive exposure to a standard dose of insecticide, owing to physiological or behavioral adaptation [44]. Resistance can be developed due to misusage or overdose usage of insecticides and selection pressure on the insect populations [45]. The question “when does the resistance emerge?” depends on the mechanism of resistance, known susceptibility, cost effectiveness and availability [45]. Various resistance mechanisms have been observed in mosquitoes: changes in their metabolism (changes in enzymes leading due rapid detoxification of insecticides), alterations in target-sites (prevention of insecticides to their target sites), penetration resistance (cuticle barrier diminishes insecticide penetration) and behavioral resistance (changes in their response to insecticidal effect) [46,47,48,49]. These mechanisms can be determined by using bioassays, biochemical assays, and molecular techniques through assessment of resistance alleles, analyzing whether metabolic enzymes are upregulated, or determination of the percent mortality rate upon exposure to a given insecticide. 

In mosquitoes, alterations of target site nerve receptors (e.g., mutations in *kdr*, *Rdl* and *Ace-1R* genes) and detoxification due to increased or modified enzyme activities (e.g., monooxygenases (P450s), glutathione-S-transferases and carboxylesterases) are the two major mechanisms responsible for insecticide resistance. According to the insecticide resistance monitoring data for 2010 to 2016, almost 70% of the assays to test resistance mechanisms included detection of the presence or absence of target-site mutations and their frequencies in WHO regions [10]. Target site alterations in mosquitoes involve knockdown resistance (*kdr*) mutations (L1014F or L1014S) in the voltage-gated sodium channel gene which causes inability of the insecticides to bind their cognate receptors [50,51,52,53,54,55]. Occurrence of *kdr* mutations causes insensitivity to pyrethroids and DDT [56,57]. A *kdr*-resistant strain of *An. gambiae* has shown to be less affected by pyrethroids than the susceptible strain [58]. In the last few decades, *kdr* resistance mutations in different mosquito populations have expanded significantly which restricts pyrethroid usage in mosquito control [59]. Another target-site mutation, the AChE gene mutation (*Ace-1R*), causes resistance to organophosphates and carbamates. In mosquitoes, a G119S mutation in the *Ace-1R* gene encoding AChE causes resistance to organophosphate and carbamate insecticides and the mutation frequency is increasing in natural mosquito populations [60,61,62,63]. A substitution mutation of alanine-to-serine/glycine (A296S/G) mutation, *Rdl*, in the second transmembrane domain of the GABA receptor subunit causes resistance to organochlorine insecticides and insensitivity in mosquitoes [35,64,65,66,67,68,69]. 

Mosquitoes have metabolic enzymes, mainly “detoxifying enzymes” that are responsible for biodegradation of insecticides and elimination of their insecticidal effects. Upon exposure to synthetic insecticides, detoxifying enzyme activity increases (due to increased gene amplification or upregulation) which result in insecticide-resistant mosquitoes [46]. Three classes of detoxifying enzymes are involved in insecticide-resistance in mosquitoes: cytochrome P450 monooxygenases (CYP), glutathione-S-transferases (GST) and carboxyl-cholinesterases (CCE) associated with pyrethroid, organochloride, and OP and carbamate resistances, respectively. Cytochrome P450 enzymes are involved in the metabolism of all four classes of insecticides. It is found that elevated levels of P450 activity resulted in pyrethroid resistant mosquito vectors [70,71,72,73,74]. Several CYPs are identified in mosquitoes and CYP overexpression is reported from insecticide resistant mosquito populations [45,59,75,76,77]. Knockdown of the CYP through the RNA-interference technique also showed that mosquitoes become sensitive to pyrethroids [78,79,80]. Glutathione S-transferases comprise a diverse family of enzymes involved in detoxification of insecticides (e.g., pyrethroids and DTT) in mosquitoes [81]. An increase in the gene expression levels of various GSTs has been detected in DDT-resistant and pyrethroid-resistant mosquitoes [82,83,84,85,86,87,88]. Additionally, a GST gene silencing study indicated an increase in the susceptibility to pyrethroid insecticide which shows that GSTs are involved in insecticide-resistance in mosquitoes [86]. Increased esterase detoxification in OP resistance has been studied most extensively in *Culex* mosquitoes [72,89]. These enzymes sequester the insecticide and interfere with its association with the target AChE by rapid binding and slow turning over of the insecticide [90]. The increase in the activity of esterases was due to overproduction of the enzymes, resulting from co-amplification of two esterase genes, *estα2* and *estβ2*, in OP-resistant individuals [91,92].

It is evident that cross-resistance causes major issues in the management of insecticide resistance through the approaches discussed above. These mechanisms can cause resistance to more than one class of insecticide (with similar mode of action) due to prolonged and intensive usage of these chemicals. For example, *Culex* mosquitoes that are resistant to a pyrethroid insecticide also show resistance to OP and other insecticides [93,94]. Pyrethroid-resistant *Anopheline* mosquitoes also show resistance to OPs due to constitutively elevated P450 levels leading to cross-resistance [95]. Moreover, insecticide resistance is genetically mediated and can be fixed in mosquito populations in such that individuals with the resistance gene will probably have a selective advantage in the presence of the insecticide [96,97]. Furthermore, mosquitoes that survive insecticide exposures possibly have the chance of passing those traits to their offspring which causes an increase in the percentage of resistant individuals in the next generations in those populations [48]. If resistance gene frequency increases in the populations, this can cause more resistant individuals to circumvent insecticidal exposures. Taken together, the emergence and spread of insecticide resistance, cross-resistance, and increased resistance gene frequencies in mosquito populations significantly effects mosquito-borne disease control and elimination and highlights the need for alternative strategies. There has been a great interest for safe and healthy biological control strategies and development of novel interventions to overcome problems associated with synthetic insecticides. Hence, extensive research for another class of insecticide for mosquito control, named “bioinsecticide”, is an ongoing process and novel natural compounds are being investigated to replace conventional synthetic insecticides. In this review, we will focus on plant-based bioinsecticides with potential activity in mosquito control.

## 4. Plant-Based Bioinsecticides

Bioinsecticides are derived from natural products, such as bioactive compounds of plants, pheromones, and from microorganisms, such as bacteria, fungi, virus, or protozoan. There are four major classes of bioinsecticides based on their nature of origin: phytochemicals, microbial pesticides, plant-incorporated protectants (PIPs), and pheromones [98] (Figure 1). They have been effectively used in pest management and generation of sustainable agricultural products [99,100]. They are less toxic, target-specific, highly effective in small quantities and biodegradable, which makes them excellent alternatives to synthetic compounds. More importantly, mosquitoes are developing resistance to synthetic compounds, a burden that needs to be resolved for successful mosquito disease control. Since biopesticides induce less insect resistance [101,102], most studies now focus on discovery of candidate natural compounds with potential effects on mosquitoes to combat mosquito-borne disease transmission. 

Plants have evolved to develop many defensive chemical compounds against pathogenic microorganisms and insects. These biologically active chemical compounds, referred to as “phytochemicals”, function as repellents, toxins, feeding deterrents, and growth regulators against insects [103]. Various parts of higher plants (leaves, roots, stems, seeds, barks, fruits, peels of fruit and resin), the whole body of little herbs, or mixture of different plants can be used for an effective plant-based insecticide. The activity of a phytochemical can change significantly depending on the plant species, plant part and its age, polarity of solvents used during extraction procedures and mosquito species [104]. Phytochemicals show their effects through targeting important cell components and affecting insect physiology in different ways; via inhibition of AChE and GABA-gated chloride channel activity, disruption of sodium-potassium ion exchange and nerve cell membrane action, blocking calcium channels, and activation of nicotinic acetylcholine receptors and octopamine receptors [105]. Moreover, phytochemicals can cause cellular destruction of epithelial cells in the midgut of mosquitoes and affect metamorphosis [106,107]. 

Several phytochemicals have been reported for their mosquitocidal activities [104,108]. These chemical compounds are mostly secondary metabolites, such as essential oils, alkaloids, phenols, terpenoids, steroids, and phenolics from different plants. Phytochemicals in plant species are diverse and discovery of those with mosquitocidal activities, which are governed by changes in expression levels of detoxifying enzymes, are of great importance to control mosquitoes. In the following sections, we provide the current knowledge on mosquitocidal plant-based compounds and their activities for a better understanding of their efficacy to prevent mosquito-borne diseases. 

## 5. Plant-Based Compounds and Mosquito Control

Plant-based compounds possess larvicidal, ovicidal and repellent activities on early or adult stages of mosquitoes, affecting nervous, respiratory, endocrine, and water balance systems. Ovicidal and larvicidal effects of many plant compounds have been extensively studied since mosquitoes are immobile at these stages and they can be efficiently eliminated before they emerge as adults. Repellent compounds are effective in keeping human hosts from mosquito bites for a blood-meal. Thus, understanding the mosquito olfactory system is vital for determination of repellent compounds. Insect repellents affect the olfactory receptor neurons via modifying or blocking its response, which in turn, elicit avoidance behavior or a change in the host-seeking behavior of mosquitoes [109,110]. There are many plant compounds with repellent activities. Essential oils, alkaloids, and aromatic compounds from various plants are commonly used for plant-based mosquito repellents [111] and they have shown to interfere with the mosquito host-seeking behavior when applied on human skin or used as indoor spraying [112]. Insecticidal and repellent activities of four major plant metabolites (essential oils, neem, pyrethrum, alkaloids) and other plant compounds (flavonoids and rotenone) are discussed in detail (Table 1).

### 5.1. Essential Oils

Essential oils have been efficiently used against a variety of pests and for crop protection in the world and they are potential alternatives to synthetic insecticides used against mosquitoes. Essential oils are very complex natural mixtures that consist of a variety of volatile molecules, which are hydrocarbons (terpenes and sesquiterpenes), oxygenated hydrocarbons and phenylpropenes (Table 1). Essential oils are synthesized in the cytoplasm and plastids of plant cells through mevalonic acid and 2-*C*-methyl-erythritol 4-phosphate (MEP) pathways, respectively [113]. Essential oils target the insect nervous system and cause neurotoxic effects through several mechanisms by inhibiting the activity of AChE, and blocking octopamine receptors and GABA-gated chloride channels [114,115]. About 90% of essential oils are composed of monoterpenes, which are determined to be active ingredients for potential plant-based larvicides and cause inhibition of AChE activity in insects [116]. Monoterpenes, such as linalool, cuminaldehyde, 1,8-cineole, limonene and fenchone, cause inhibition of AChE and accumulation of acetylcholine in synapses and state of permanent stimulation, which results in ataxia [117,118]. According to Hideyukiu and Mitsuo [119], a mixture of monoterpenoids is a more potent inhibitor of AChE than single monoterpenoid application and acts synergistically.

**Table 1:** An overview of insecticidal activity and mechanism of action of various plant-based compounds against mosquito species.

Type of Botanical Product	Plant Family	Activity	Mechanism of Action	Mosquito Species	References
Essential Oils Monoterpenes: linalool, cuminaldehyde, 1,8-cineole, limonene, fenchone, eugenol, γ-terpineol, cinnamic alcohol, geraniol, β-citronellol, *P*-menthane-3,8 diol, α-pinene, β-pinene, *p*-cymene, thymol, terpinolene, camphor, citronellal, sabinene, carvacrol Sesquiterpenes: guaiol, α-bisabolol, α-cadinol, germacrene D, β-caryophyllene, nootkatone Diterpenoids: diterpene alcohol, phytol Aromatic phenol Coumarin	Anacardiaceae Annonaceae Apiaceae Asteraceae Geraniaceae Lamiaceae Lauraceae Poaceae Rutaceae Myrtaceae Verbenaceae	larvicidal, pupaecidal, ovicidal, adulticidal, repellent, antifeedant, growth and reproduction inhibitors	Inhibition of AChE Blockage of GABA-gated chloride channels Agonist of octopamine receptors	*Cx. pipiens pallens* *Cx. quinquefasciatus* *Cx. pipiens biotype molestus* *Ae. aegypti* *Ae. albopictus* *An. gambiae* *An. stephensi*	[120,121,122,123,124,125,126,127,128,129,130,131,132,133,134,135,136,137,138,139,140,141,142,143,144,145,146]
Neem oil azadirachtin, meliantriol, salannin, desacetyl salannin, nimbin, desacetyl nimbin, nimbidin, nimbolide, deacetylgedunin, gedunin, 17-hydroxyazadiradione, deacetylnimbin	Meliaceae	repellent, ovicidal, larvicidal, feeding deterrence, fecundity suppression, toxicity, growth regulation, oviposition deterrence	growth inhibitors, hormonal disruption (ecdysone blocker), molting aberrations, interference with phagostimulants	*An. gambiae* *Ae. aegypti* *Ae. albopictus* *An. stephensi* *Cx. quinquefasciatus*	[147,148,149,150,151,152,153,154,155,156,157,158,159,160,161,162,163,164,165]
Pyrethrum esters of chrysanthemic acid: pyrethrin I, cinerin I, jasmolin I esters of pyrethric acid: pyrethrin II, cinerin II, jasmolin II	Asteraceae	repellent, knock-down effect, blood-feding inhibition	voltage-gated sodium channel modulator	*An. gambiae*	[166,167,168,169,170]
Alkaloids alpha-solanin ricinine pyridine nicotine diterpene nornicotine anabasine	Berberidaceae Fabaceae Solanaceae Ranunculaceae Euphorbiaceae	repellent, larvicidal	interfering with cellular and physiological functions, inhibition of AChE activity, regulation of hormone activity, toxicity, agonist of acetycholine receptor	*Ae. aegypti* *An. arabiensis* *An. gambiae* *Ae. albopictus* *An. stephensi* *Cx. pipiens*	[171,172,173,174,175,176,177,178,179,180,181]
Flavonoids	Zingiberaceae	larvicidal	inhibition of AChE, degradation of cell membranes acting as stomach poisons	*Ae. aegypti*	[182,183,184]
Rotenone	Fabaceae	larvicidal	inhibitor of the cellular respiration system	*Ae. aegypti*	[185]

The octopaminergic system of insects is another target for essential oils that block octopamine receptors and cause acute and sub-lethal behavioral effects on insects. The increase in cyclic AMP levels, induced upon binding of octopamine to octopamine-receptors, can be inhibited by a mixture of essential oils (eugenol, γ-terpineol and cinnamic alcohol). Moreover, octopamine receptor binding is significantly reduced with low doses of eugenol alone [120,121]. Another possible target for essential oils is ligand-gated chloride channels. Essential oils consist of monoterpenes, such as linalool, methyl eugenol, estragole, citronellal, inhibit GABA-gated chloride channels by binding at the receptor site and increase the chloride anion influx into the neurons, which lead to hyper-excitation of the central nervous system, convulsions, and finally death of insects [122,123].

Many plant oils possess ovicidal, larvicidal, pupaecidal and repellent activities against various mosquito species, some of which will be discussed below. Essential oils of plants from the Lamiaceae, Poaceae, Rutaceae and Myrtaceae families are well-known for repellent activity [103]. Essential oils obtained from citronella, lemon and eucalyptus are commercially available and recommended by the U.S. Environmental Protection Agency (US EPA) as repellent ingredients for application on the skin because of their low toxicity. For example, *P*-menthane-3,8 diol (PMD) is an active component of the lemon eucalyptus plant and responsible for the repellency in mosquitoes [124]. 

Most of the monoterpenes and sesquiterpenes of essential oils are known with repellent activities [125]. Among monoterpenes, α-pinene, γ-pinene, *p*-cymene, eugenol, limonene, thymol, terpinolene, citronellol, camphor and citronellal are responsible for mosquito repellency [126,127]. Representative molecules of sesquiterpenes are guaiol, α-bisabolol, α-cadinol, germacrene D, β-caryophyllene and nootkatone. β-caryophyllene is known to exhibit strong repellent activity against *Aedes* mosquitoes [126]. Repellent and larvicidal activities of monoterpenes from the essential oils of *Thymus* plant against *Cx. pipiens pallens*, *Cx. quinquefasciatus*, and *Cx. pipiens* biotype *molestus* have been determined [128,129,130]. Larvicidal activities of phenolic terpenes, such as thymol and carvacrol, of *Satureja* species were observed against *Cx. pipiens* biotype *molestus* [131]. Moreover, repellent and larvicidal activities of carvacrol were determined in the field trials against *Ae. albopictus* mosquitoes in Bologna (Italy) [132]. *Cinnamomum osmophloeum* and *Carum copticum* essential oils had larvicidal activity against *Cx. quinquefasciatus* and *Cx. pipiens*, respectively [107,133]. Toxicity of β-citronellol, geraniol and linalool from *Pelargonium roseum* essential oil was also detected in *Cx. pipiens* [134]. High larvicidal and pupaecidal activities of essential oils from *Cinnamomum verum*, *Citrus aurantifolia*, *Cuminum cyminum*, *Syzygium aromaticum*, *Laurus nobilis*, *Lippia berlandieri* and *Pimpinella anisum* were reported from *Cx. quinquefasciatus* [135]. *Artemisia absinthium* essential oils also showed toxic effects against larval populations of *Aedes*, *Anopheles*, and *Culex* mosquitoes [136]. Essential oils isolated from *Tagetes lucida*, *Lippia alba*, *Lippia origanoides*, *Eucalyptus citriodora*, *Cymbopogon citratus*, *Cymbopogon flexuosus*, *Citrus sinensis*, *Swinglea glutinosa*, and *Cananga odorata* plants showed larvicidal activities on *Ae. aegypti* larvae [137]. Oviposition deterrence and ovicidal activity of some of essential oils, peppermint oil, basil oil, rosemary oil, and citronella oil from *Mentha piperita*, *Ocimum basilicum*, *Rosmarinus officinalis*, *Cymbopogon nardus* and *Apium graveolens* were also reported in *Ae. aegypti* [138]. Manh et al. [139] also showed toxicity of essential oils from *Eucalyptus* and *Cymbopogon* aromatic plants to the larvae of *Ae. aegypti*. Essential oils also cause toxicity at different developmental stages and have repellent activities against adult *Anopheles* mosquitoes [140]. Essential oils extracted from *Cymbopogon proximus*, *Lippia multiflora* and *Ocimum canum* had larvicidal and ovicidal activities against *An. gambiae* and *Ae. aegypti* mosquitoes [141]. Besides monoterpenes and sesquiterpenes, phytol (a diterpene alcohol) and coumarin (an aromatic phenol) were both determined to be responsible for the biting deterrence effect in *Ae. aegypti* [142].

Repellent activity of essential oils is generally attributed to individual chemical compounds, but synergistic effects of plant metabolites have been observed when the effect of an active compound is enhanced by other major compounds or modulated by minor compounds. The efficacy of the major compounds is enhanced by minor compounds through different mechanisms, which may cause higher bioreactivity compared to isolated compounds of essential oils. The synergistic effect is also observed with mixture of oils. The synergistic action of the major compounds in essential oils results in higher repellent and larvicidal activity and toxicity to insects [140,143,144,145]. A combination of blends assayed on *An. gambiae* mosquitoes indicated that blends of oils showed higher repellency compared to the individual oil used [146]. It has been also reported that essential oils composed of a mixture of active components might reduce resistance in mosquito population by acting at different target sites or with a different mode of action [139]. 

### 5.2. Neem

Neem-based insecticides are extensively used for protection against various pests all over the world. Neem trees, *Azadirachta indica*, is a member of the Meliaceae family and are originated from India and distributed throughout all South- and Southeast-Asian countries, including Pakistan, Sri Lanka, Thailand, Malaysia, and Indonesia [147]. The main product of the neem is the oil extracted from the seeds and contains at least 100 active compounds, including azadirachtin, meliantriol, salannin, desacetyl salannin, nimbin, desacetyl nimbin, nimbidin and nimbolides [148]. Limonoids are the major active compound of the neem oil and act as an insect growth inhibitor. Azadirachtin is a triterpenoid and highly oxidized limonoid, one of the most potent active compounds of the neem extract and found in higher concentrations (0.2–0.6%) in the seeds of the neem compared to other parts of the neem tree [149,150]. Various isomers of azadirachtin (azadirachtin A to G) were identified and azadirachtin A and B isomers are the most abundant isomers in the plant tissues. In addition, azadirachtin A is the most active biological ingredient which shows insecticidal activity compared to the other analogs [151,152,153]. 

Generally, neem-based products are effective in the juvenile stages of insects. Azadirachtin is structurally similar to insect hormones known as ecdysones that are involved in the process of metamorphosis. The main mechanism of action of azadirachtin is to impair the homeostasis of insect hormones by interfering with the endocrine system. Azadirachtin acts as ecdysone blocker and causes severe growth and molting aberrations by affecting ecdysteroid and juvenile hormone titers [154]. The feeding deterrent activity of azadirachtin is mediated through azadirachtin’s interference with phagostimulants that are important in normal feeding behavior of mosquitos [155].

Neem-based biopesticides have a wide range of effects against insects, such as re-pellency, feeding deterrence, ovicidal activity, fecundity suppression, toxicity, insect growth regulation, deterrence of egg-laying, disruption of growth and reproduction, and inhibition of metamorphosis [156,157,158,159,160]. Larvicidal activity of the neem oil has been reported in controlling mosquito larvae in different breeding sites under natural field conditions [161]. Ayinde et al. [162] reported the repellent and larvicidal potential of the emulsified neem seed oil formulation as a suitable alternative for commercially available insecticides against *An. gambiae* in Nigeria. Oils of neem and karanj were also found to have larvicidal, ovicidal and oviposition deterrent activities against *Ae. aegypti* and *Ae. albopictus* mosquitoes [163]. The effects of the neem limonoids azadirachtin, salannin, deacetylgedunin, gedunin, 17-hydroxyazadiradione and deacetylnimbin were analyzed, and azadirachtin, salannin and deacetylgedunin showed the highest larvicidal activity against *An. stephensi* [164]. Larval mortality and repellent activity were also achieved from neem essential oils against *An. gambiae* [162]. A neem extract, neemarin, also showed significant mortality rates at larvae, pupae, and adult stages of *Cx. quinquefasciatus* and *An. stephensi*, where the former showed lower mortality rates [165].

### 5.3. Pyrethrum

Pyrethrum is a plant-based insecticide obtained from flower heads of *Tanacetum cinerariifolium*. Pyrethrum extract is composed of six active ingredients derived from esters of chrysanthemic acid: pyrethrin I, cinerin I, and jasmolin I, and esters of pyrethric acid: pyrethrin II, cinerin II, and jasmolin II [166]. They target the nervous system of insects and cause neurotoxic effects through blocking the voltage-gated sodium channels in nerve axons, thereby cause hyperactivity and convulsions by a rapid knockdown effect [167]. The mode of action of pyrethrins is similar to that of DDT and many synthetic organochlorine insecticides. Thus, pyrethrins can be alternatively used instead of organophosphates and organochlorides. While it is less toxic to mammals, it has higher toxicity to fish and aquatic invertebrates. When used together with a conventional synergist, such as piperonyl butoxide (PBO), their activity is increased and harmful effects to non-target organisms are reduced [168]. The usage of natural pyrethrins in mosquito control is supported with the finding that pyrethrum had knock-down effect, repellency, and blood-feeding inhibition in pyrethroid-resistant *An. gambiae* strains [169]. Electroantennogram responses of pyrethrum in *Ae. aegypti* and *An. gambiae* mosquitoes were detected while no response is observed in maxillary palps, indicating that the repellency effect of pyrethrum is mediated by the olfactory systems of mosquitoes [170]. Moreover, the molecular mechanism of pyrethrum repellency was investigated and a synergistic mechanism involving dual activation of olfactory repellency pathways and voltage-gated sodium channels has been determined [170].

### 5.4. Alkaloids

Alkaloids are nitrogen-containing natural products found in bacteria, fungi, animals, and plants. They are commonly isolated from plants and found in large quantities in many members of the Berberidaceae, Fabaceae, Solanaceae, and Ranunculaceae families. The alkaloids obtained from these plants are used extensively in conventional insect repellents [171,172,173]. The mode of action of alkaloids varies depending on the type of alkaloids and interferes with major cellular and physiological functions by affecting AChE receptors in the nervous system, regulating hormonal activity, and causing toxicity [174]. Alkaloids are not volatile like essential oils. However, they could be used as repellents against mosquitoes by burning plants to generate an insecticidal smoke that repels insects and directly causes toxicity [124]. In *Ae. aegypti*, the inhibitory effect of natural alkaloids on AChE activity was determined by using molecular docking studies. Among the 25 different alkaloids tested, alpha-solanine has been found to fit into the AChE1 binding pocket and potentially be the best inhibitor of AChE1 [175]. 

Extracts of the castor bean (*Ricinus communis*, Euphorbiaceae) contain the alkaloid ricinine and have a strong insecticidal effect. It showed strong larvicidal activity against larvae of *An. arabiensis* [176]. Additionally, pyridine alkaloid from *R. communis* showed bioactivity against *An. gambiae* larvae and adults [177]. The larvicidal activity of alkaloids against *Ae. albopictus*, *Cx. pipiens pallens* and *Ae. aegypti* has also been determined [178,179]. Alkaloid from *Arachis hypogaea* plant also had larvicidal toxicity against *An. stephensi* and *Ae. aegypti* mosquitoes [180].

Nicotine is an alkaloid derived from tobacco plant (*Nicotiana tobacco*) that mostly consists of phenolic compounds, such as nicotine and diterpene. Nicotine, nornicotine and anabasine mimic the neurotransmitter acetylcholine, which causes symptoms similar to organophosphate or carbamate insecticides [160]. Extracts of tobacco leaves were mixed with bio-oil and high repellent activity was observed against *Ae. aegypti* [181]. Furthermore, nicotine has been found to be the most dominant compound among the other active compounds of the repellent mixture, including nicotine, d-limonene, indole, and pyridine. In addition, the repellent compound was harmless to human skin as confirmed by sensitivity tests on volunteers. 

### 5.5. Other Plant Compounds

Besides the most common plant-based bioinsecticides mentioned above, there are other natural plant metabolites that show insecticidal properties. Among them, flavonoids elicit larvicidal activity by inhibiting AChE in mosquito larvae [182]. They could also act as respiratory inhibitors and result in the disturbance of the larval respiratory system. Alkaloids have multiple effects including inhibition of the AChE enzyme, degradation of cell membranes, and they may act as stomach poisons [182]. It has been shown that flavonoid and alkaloid components of bangle rhizome extract from *Zingiber montanum* act differently against *Ae. aegypti* [183]. Flavonoids from *Derris trifoliata* extract also exhibited larvicidal activity against *Ae. aegypti* [184]. Rotenone is an isoflavonoid extracted from roots and stems of *Derris* (*Derris elliptica*, *Derris involute*), *Lonchocarpus* (*Lonchocarpus utilis*, *Lonchocarpus urucu*) and *Tephrosia virginiana* [160]. It has long been used as a biopesticide due to less harmful effects to the environment. Rotenone has the potential to be used as a larvicide to control mosquitoes and interferes with the cellular respiration system of insects and prevents energy production [185]. 

## 6. Assessment of Plant-Based Bioinsecticide Efficacy in Mosquito Control

It is important that inherent activity of candidate bioinsecticides should be assessed before they can be effectively used against mosquito populations. The World Health Organization has established methods to screen the efficacy and field application acceptability of new compounds as potential mosquito larvicides and adulticides (for IRS and ITNs); they are laboratory studies, small-scale and large-scale field trials [186,187,188]. Laboratory studies focus on determination of biopotency, efficacy, residual activity, irritant or repellent properties, diagnostic concentration, and possible cross-resistance of candidate larvicides or adulticides. In laboratory bioassays, mosquito larvae are exposed to various concentrations of larvicides, and a mortality rate based on lethal concentration (LC) of the larvicide for 50% and 90% mortality (LC50 and LC90) or for 50% and 90% inhibition of adult emergence (IE50 and IE90) is recorded. LC values are determined and can then be compared with the LC50 or LC90 values of other insecticides to assess the activity of the compound as “sufficiently effective”. For adulticides, LC is determined by tarsal contact to treated papers. The “time to first take-off” (FT) for the 50% and 90% of the mosquitoes to take off (FT50 and FT90) after exposure to treated substrates are measured to determine the irritant or repellent activity of an adulticide. Insecticide-treated nets are used for bioassays of adult mosquitoes to determine the efficacy and residual activity of different dosages of the candidate compounds. Moreover, efficacy and wash-resistance of ITNs against susceptible mosquito species should be determined using standard WHO cone bioassays or tunnel tests [188]. The efficacy criteria for cone bioassays are ≥80% mortality or ≥95% knock-down, and for the tunnel test, it is ≥80% mortality or ≥90% blood-feeding inhibition. Candidate larvicides and adulticides are also tested against multi-resistant mosquito strains and a susceptible reference strain to assess the cross-resistance and, if detected, biochemical, immunological, and molecular methods are used to determine the mechanism of resistance [189]. 

Once candidate compounds are selected from laboratory tests, they are subjected to small-scale field testing in natural breeding sites (such as drains sewage water tanks, ponds, rice plots, etc.) or under simulated field conditions (artificial containers filled with water, experimental huts). Larvicidal efficacy is determined by the level of inhibition of emergence of adults and the percentage reduction in larval and pupal densities, while adulticidal efficacy can be assessed in terms of mortality, residual effect, deterrence, blood-feeding inhibition and induced exophily. These trials elucidate efficacy of candidate compounds against different mosquito species in different breeding sites, determine optimum field application dosage of the compound and possible impact on the mosquito behavior. Abiotic parameters that may influence the efficacy of the product and effect on non-target organisms can also be observed. Those larvicides and adulticides that show promise in small-scale field trials should be validated in larger-scale field trials against natural mosquito populations in natural breeding habitats using optimum field dosages. At this stage, the storage, handling, and application of the insecticide formulation should be considered for proper functioning of application and dispersal of the bioinsecticide in natural ecosystems. 

There are also potential limitations to the efficacy of bioinsecticides, such as environmental conditions, mosquito fitness, mosquito resistance as well as the parts of the plants used, solvents used in extraction steps, insecticide dose and exposure time [190,191]. These effects should be considered for successful assessment of novel bioinsecticides in mosquito control. While efficacy tests provide promising information on possible mosquitocidal effects, new compounds from plant origin, the identification of actual active ingredient for efficacy and their mode of action are still waiting to be resolved.

## 7. Effective Use of Plant-Based Bioinsecticides in Resistant Mosquito Populations

Most of the bioinsecticides are now effective alternatives to chemical insecticides and have become an integral part of the integrated mosquito management (IMM) programs because the development of resistance to bioinsecticides is low due to their multiple mode of actions [192,193]. The synergic mixture of the active compounds in plant extracts also minimizes resistance development [167]. However, resistance already developed to extensively used chemical insecticides is a major problem that limits the success rate of novel bioinsecticides against mosquito populations. Insecticide resistance should be reduced or reverted (which takes time) in order to apply new and effective bioinsecticides in resistant populations. Surveillance of mosquito resistance and effective resistance management strategies should be routinely conducted to determine the levels, mechanisms, and geographic distribution of resistance in field populations of mosquitoes for increasing efficacy of bioinsecticides [44]. Moreover, proper application technologies should be considered as they greatly influence the bioinsecticide efficacy. 

Surveillance of resistance development to many different insecticides are determined by dose-mortality bioassays, the World Health Organization tube testing, and Centers for Disease Control and Prevention (CDC) bottle bioassay for mosquitoes [11,44,194,195]. In the dose-mortality assay, the resistance ratio (RR) is determined in a susceptible population to monitor changes in resistance over time. The RR is calculated from LC50 values of the field and susceptible populations, in which an RR lower than five indicates susceptibility or low resistance and an RR value higher than ten indicates high resistance. In the WHO tube testing, the insecticide susceptibility status of the selected mosquitoes is evaluated through susceptibility tests measuring the mortality rate twenty-four hour after exposure [44]. A mortality rate lower than 98% indicates occurrence of resistance and should be confirmed with biochemical and molecular analysis. A mortality rate less than 90% confirms the existence of resistant genes in the tested mosquito populations. The CDC bottle bioassay is a measure of insecticide effectiveness, where diagnostic doses (DDs) and diagnostic times (DTs) are determined for candidate compounds using susceptible mosquitoes prior to testing in field mosquito populations. The DD is a measure of insecticide dose that kills 100% of susceptible mosquitoes within a certain period of time (DT). A mortality rate lower than 97% is an indication of resistance that needs to be confirmed, and below 80% suggests strong resistance at the recommended DT. The DD and DT values for some active ingredients are available for *Anopheles* and *Aedes* mosquito populations and these parameters should be defined for a particular insecticide and mosquito population [195]. 

It is evident that no single strategy is effective enough to solve insecticide resistance of mosquitoes. According to the WHO [44], one strategy to prevent the resistance problem is rotational usage of different classes of bioinsecticides with different modes of action. There are several new plant-based larvicides with different modes of action (discussed in Section 5) and they could be good alternatives for mosquito control in larval stages. Additionally, multiple interventions that affect different stages of mosquitoes (such as larvae and adults) can be used together to manage insecticide resistance. It is also suggested that different classes of insecticides with different modes of action can be used in neighboring geographic locations. To successfully implement these strategies, knowledge of the mode of action of the novel bioinsecticide is essential. The resistance mechanism developed by the local population of mosquitoes should also be determined to reduce cross-resistance effects. 

RNA interference (RNAi) mediated loss-of-function technique has been proposed for pest management programs [196,197] and to study insecticide resistance [198]. Genes responsible for resistance development in insects (e.g., genes for DDT or pyrethroid resistance) can be identified and used as a target for the development of novel RNAi based insecticides. Several delivery methods including nonmicrobial and microbial are used routinely to induce RNAi in mosquito larvae [199]. Nonmicrobial delivery methods consist of soaking, injection, nanoparticles and dehydration and rehydration. Although soaking and injection methods are used as excellent tools in RNAi research, they have no application in the field. Soaking, injection methods and nanoparticles have been effectively used to introduce dsRNA into first-instar *Ae. aegypti* larvae [200] and fourth instars of *Ae. aegypti* [201]. In mosquitoes, a chitosan/dsRNA-based nanoparticle has also been used in feeding the larvae of *An. gambiae* mosquitoes which led to successful gene silencing of two chitin synthase genes and increased susceptibilities to DTT [202]. Such an RNAi-based bioinsecticide can be potentially used as an effective strategy to enhance the efficacy of new bioinsecticides for mosquito control.

Another technology used for the manipulation of insect behavior is “Specialized Pheromone and Lure Application Technology (SPLAT)”. SPLAT is a chemical controlled-release emulsion technology, and it has been used as an alternative management strategy to target the aquatic life stages of mosquitoes [203]. SPLAT emulsions can be formulated by using a variety of compounds, such as sex pheromones, attractants, repellents, phagostimulants and insecticides. SPLAT consists of both aqueous and non-aqueous components. The aqueous component of the SPLAT emulsion is involved in the liquid property of the product and evaporates within 3 h upon application. The non-aqueous component of the emulsion is the controlled-release device that releases active ingredients (e.g., semio-chemical or pesticides) at a controlled rate for 2 weeks to 6 months by protecting the active ingredients from environmental, chemical, and biological degradation. It has been reported that combination of attractant and larvicidal agents in a single formulation and biodegradable matrices causes significant increase in larval mosquito mortality, specifically *Cx. quinquefasciatus*, compared to formulations consisting of larvicidal agents alone in semi-field trials (e.g., large-screened greenhouse and emulating field conditions) [204]. The major benefits of this technology are a timely-manner release of both pheromone and insecticide, reduced insecticide resistance, and persistence in the field [203]. 

## 8. How to Improve Plant-Based Bioinsecticide Efficacy in Mosquito Control Strategies?

Synthetic chemicals used to control mosquitoes are now causing serious health problems and, more importantly, resistant mosquitoes that lead to search for more effective, healthier, safer, and eco-friendly natural solutions. Phytochemicals derived from plant resources are excellent targets to search for bioactive compounds because plants synthesize these chemicals naturally in response to their environment (such as against insect predators and microbial attacks), thus, plants are indeed natural insecticide sources. While searching the literature for plant-based compounds, we have encountered a tremendous number of efforts to identify and evaluate compounds that could have potential mosquitocidal activity with negative impact on mosquito physiology at different development stages. Since phytochemicals have multiple modes of action and exert their effects on multiple target sites in insects, their efficacy can be enhanced when used as a blend (e.g., mixture of oils) against mosquitoes. In addition, insects are more likely to develop resistance to a single chemical compound rather than a mixture of compounds. Thus, a combinatorial usage of phytochemicals would limit development of resistance in mosquitoes. Phytochemicals have short residual half-life which could be advantageous when synergistically used together with other biological control agents [205]. It is encouraging that these features of phytochemicals make them alternative natural solutions for the development of suitable products to interfere with the mosquito–host interaction and reduce disease transmission. 

Among the phytochemicals, essential oils are extensively studied and their repellent activities against mosquitoes makes them favorable natural chemicals. However, they are volatile compounds, and this causes issues in their long-term applications in mosquito control. In recent years, new technologies, such as microencapsulation and nanoemulsion, have been used to overcome this problem by enhancing the duration and efficacy of essential oils [140]. Since ITNs are one of the major intervention methods to control mosquitoes, the incorporation of plant-based insect repellents in fabrics seems a prompt and alternative way to provide safer protection against mosquito bites. Fabrics treated with microencapsulated citronella essential oil have been reported to provide higher repellent activity and longer lasting protection, up to three weeks, against insects compared to the fabrics sprayed with ethanol solution of the essential oil [206]. Grancaric et al. [207] also reported that microencapsulated immortelle oil had the highest repellent efficacy against *Ae. aegypti* compared to immortelle oil alone on cotton samples. In another study, microcapsules composed of two biopesticides, namely citronella essential oil and citriodiol, were prepared and applied to cotton textiles using a variety of techniques. As a result, citriodiol-treated cotton fabrics had a prolonged durability, and 100% repellent activity for more than 30 days after its application [208]. Additionally, encapsulation of citronella oil into microcapsules of poly ε-caprolactone has been considered as an effective and sustained release system with potential application in protection against mosquitoes [209]. Encapsulated citronella oil nanoemulsions prepared by high pressure homogenization at varying amounts of surfactant and glycerol were tested for mosquito repellency. It has been shown that increasing concentration of glycerol and surfactant improved the stability of the emulsion causing prolonged mosquito protection [210]. These results clearly indicate that through microencapsulation and nanoemulsion formulation technologies, effective and longer usage of essential oils on cotton fabrics or ITNs can be achieved. 

Neem-based insecticides can also be effectively used for the control of mosquitoes. They are considered more eco-friendly than synthetic insecticides and are less prone to induce resistance because of their multiple modes of action on insects. Another advantage of neem oil formulations is that it causes mortality at relatively low concentrations making them potential alternatives to synthetic insecticides in the control of malaria vectors. Microencapsulation of neem seed oil and karanja oil has been used for the control of larvae of *Ae. aegypti* [211]. The major drawback of using neem oil is that its dosage should be considered when applied in the field because neem can cause risks to non-target organisms at higher doses. 

Natural pyrethrins are now considered as a potential alternative to synthetic DTT and can overcome hazardous effects of pyrethroids. However, they have major drawbacks which include their high instability and quick degradation in the presence of sunlight. Stability concerns and short duration of their knockdown effect cause inadequate field applications against mosquito populations during the day [212]. However, the application of pyrethrin-based insecticides after sunset against *Culex* and *Anopheles* has shown a decrease in mosquito populations and protection against non-target insects [213]. Pyrethrins are also more effective when used with a synergist. They can be easily degraded before having an impact on mosquitoes, thus should be applied with a synergist of non-synthetic origin. Since pyrethrin-based chemicals are detected via mosquito olfactory organs and processed through olfactory signal transduction mechanisms, pyrethrin-based repellent molecules should be developed and implemented in order to interfere with the host-seeking behavior of mosquitoes for an effective reduction in disease transmission.

Despite our increasing knowledge on plant-based bioactive compounds and their multiple mode of actions on insects, a few of them, such as essential oil-based and neem-based insecticides, have been commercially available for pest management [205]. One of the reasons that causes their limited usage in the field is the formulation problem to overcome phytotoxic effects. The chemical composition of each compound should be formulated in such a way that it should be bioactive to target insects and non-toxic to non-target organisms. In addition, formulation of plant-based bioinsecticides should ensure that it can be produced in large quantities through biomass production of plants and administered in recommended dosages to minimize toxic effects, and biological activity can be maintained for longer shelf-life. As discussed above, microencapsulation and nanoemulsion technologies have benefits in solving formulation issues of phytochemicals. A new formulation in the form of tablets containing a lectin preparation showed mosquitocidal activity against different developmental stages of *Ae. aegypti* mosquitoes, and this formulation method is proposed as a new control strategy for *Ae. aegypti* populations [214]. Phytochemicals break down rapidly and this causes a need for continuous and more frequent applications in the field for a satisfactory impact on mosquito control. Further studies are needed with the implementation of new methods for the development of effective bioinsecticides from other plant-based bioactive compounds.

## 9. Conclusions

Mosquitoes are important vectors of devastating diseases, and their hazardous effects are far beyond eradication. The occurrence/reoccurrence of mosquitoes in endemic, non-endemic, and new regions of the world has led to extensive use of synthetic chemicals to control transmission of mosquito-borne diseases. With the increase of resistant mosquitoes and toxicity issues to target and non-target organisms, safer, biodegradable, target-specific alternatives have been considered to replace conventional mosquito control strategies. Phytochemicals have gained importance to overcome mosquito control problems as being considered natural, environmentally safe, less toxic, inexpensive, and, more importantly, less prone to mosquito resistance. Variety of plant extracts have been reported to have mosquitocidal or repellent activity against mosquito vectors, mostly depending on laboratory assays, but there are limitations for their efficacy and applicability in the field. Problems associated with their formulation and commercialization, non-standardization in evaluation of their bioactivities, and their persistence for longer durations should be resolved for development of effective and sustainable methods for their usage. There is no doubt that there are more bioactive compounds that require exploring, and future research should focus on searching for plant-based products with the ultimate goal of deploying them as a reliable remedy to control mosquito populations and mosquito-borne diseases.

## Data Availability

Not applicable.
